# Practitioners' Bookshelf

**Published:** 1891-10-10

**Authors:** 


### PRACTITIONERS' BOOKSHELF.

### INTUBATION OF THE LARYNX.*

It daily becomes more essential that every medical practitioner should be acquainted with this operation, which O'Dwyer has practically invented and introduced. The little work before us consists chiefly of papers by the author, published in the *Illustrated Medical News* in 1889, with additions, and brought up to date.

The instruments and the method of performing the operation are clearly set forth, and with the help of this book and some preliminary practice on the cadaver, there should be no great difficulty in performing the operation on an emergency. We must, however, take exception to the opinion of the author that the practice of some surgeons in performing the operation without the use of the gag offers no advantages. It certainly does increase the difficulties somewhat, if the gag be dispensed with, but the dyspnœa is often greatly increased by gagging, in fact children have actually died from asphyxia, as a result of the increased obstruction to respiration.

The author may be congratulated on his handy and practical guide.

### ON STERTOR, APOPLEXY, AND THE MANAGEMENT OF THE APOPLECTIC STATE.†

There are many of the commoner conditions and ailments of patients which have always been relegated to a minor place in medical literature, partly because their pathology seems simple, partly because they are indefinite "states" rather than pathological entities, of these neglected states apoplexy is one. The name of Dr. Bowles has so long been associated with the investigation of stertor and apoplexy, that we may naturally expect to find a contribution from him on this subject full of fresh views and much-needed practical advice. The author has most carefully investigated the cause of stertor in apoplexy, and he points out that the presence or absence of this sign generally depends on whether the patient is or is not lying on his back, but that if it is present when the patient is lying on his side, it is generally due to the chin being too near the sternum, or to some local obstruction in the larynx or fauces. The author's remarks are rendered more clear and forcible by some well-executed diagrams. Few medical practitioners can read this little work without learning much that is useful.

* "Intubation of the Larynx," by James B. Ball, M.D. Lond., Physician to the West London Hospital. Illustrated; 8vo. pp. 84. London: H. K. Lewis, 1891.

† "On Stertor, Apoplexy, and the Management of the Apoplectic State." By Robert L. Bowles, M.D., F.R.C.P. London, Baillière Tindall and Cox, 1891.

